# Comprehensive Review of Recent Advances in Chiral A-Ring Flavonoid Containing Compounds: Structure, Bioactivities, and Synthesis

**DOI:** 10.3390/molecules28010365

**Published:** 2023-01-02

**Authors:** Changyue Zhang, Yanzhi Liu, Xiaojing Liu, Xiaochuan Chen, Ruijiao Chen

**Affiliations:** 1 Medical Laboratory of Jining Medical University, Jining Medical University, Jining 272067, China; 2 School of Pharmacy, Binzhou Medical University, Yantai 264003, China; 3 College of Basic Medicine, Jining Medical University, Jining 272067, China; 4 Gan&Lee Pharmaceticals, Beijing 101109, China; 5 Key Laboratory of Green Chemistry and Technology of Ministry of Education, College of Chemistry, Sichuan University, Chengdu 610064, China

**Keywords:** flavonoids, chiral A-ring-containing flavonoid, bioactivity, synthesis

## Abstract

Flavonoids are a group of natural polyphenolic substances that are abundant in vegetables, fruits, grains, and tea. Chiral A-ring-containing flavonoids are an important group of natural flavonoid derivatives applicable in a wide range of biological activities such as, cytotoxic, anti-inflammatory, anti-microbial, antioxidant, and enzyme inhibition. The desirable development of chiral A-ring-containing flavonoids by isolation, semi-synthesis or total synthesis in a short duration proves their great value in medicinal chemistry research. In this review, the research progress of chiral A-ring-containing flavonoids, including isolation and extraction, structural identification, pharmacological activities, and synthetic methods, is comprehensively and systematically summarized. Furthermore, we provide suggestions for future research on the synthesis and biomedical applications of flavonoids.

## 1. Introduction

**Figure 1:**
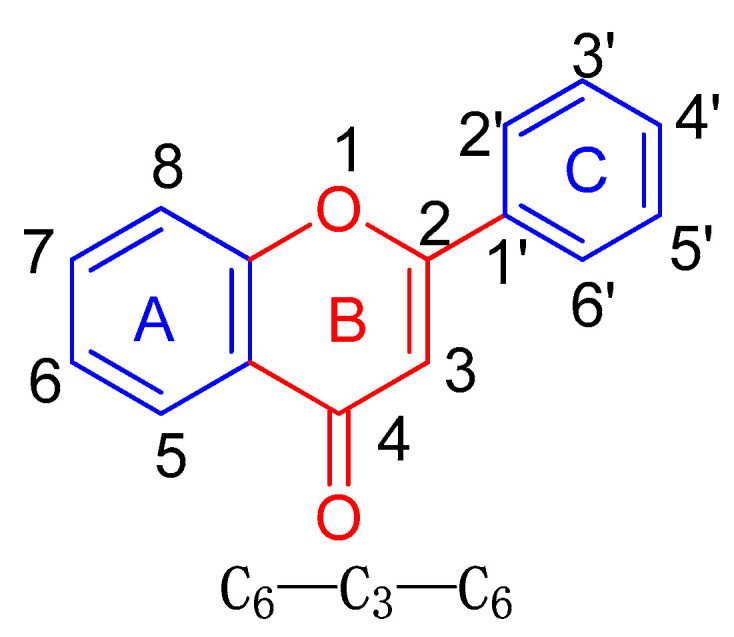
Basic skeleton of flavonoid compounds.

Flavonoids are a class of secondary metabolites produced by long-term natural selection of plants, and they play an essential role in plant growth and development [1,2], flowering and fruiting [3], anti-inflammatory [4,5], and anti-disease [6,7,8,9,10]. Flavonoids are structurally diverse and have different biological and pharmacological applications, such as antioxidant [11,12,13,14], hypotensive [15,16,17,18,19], antibacterial [20,21,22,23], antiaging [24], anti-fatigue [25,26,27], antiviral [28,29,30], and antitumor [31,32,33,34,35]. Flavonoids are important in disease prevention, treatment, and the development of new drugs because of their low toxicity and broad biological application. Flavonoids, whose structures are shown in (Figure 1), currently refer to a wide range of natural products with two benzene rings (A and C rings) linked by a central triple carbon to form a C6-C3-C6 primary parent nucleus. Among them, there is a distinct class of flavonoid compounds, which have an acetate (ester) or lactone group attached to the 5th position of their A-ring and are chiral in nature. The unique characteristics of these backbone molecules can be used as a marker of chemical differentiation [36]. These compounds have promising biological activities, such as tyrosine kinase inhibition [37], antituberculosis [38], glucose transport inhibitory [39], and so on. Thus, these flavonoids and analogs are a potential agent for the treatment of cancer and other diseases. In this paper, the structures, synthesis and bioactivities of chiral A-ring flavonoid compounds were summarized for these unique structures in the following aspects.

## 2. Isolation and Structural Elucidation of Chiral A-Ring Flavonoid-Containing Compounds

### 2.1. Cryptocaryone

**Figure 2:**
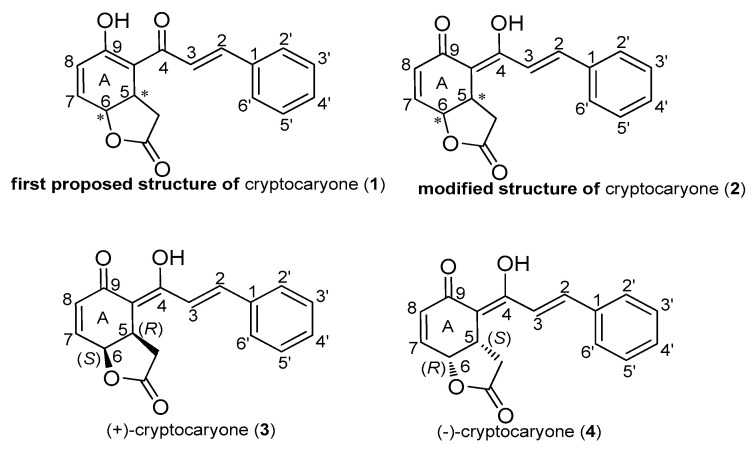
Chemical structure of cryptocaryone.

Cryptocaryone was first isolated from the roots of a thick-shelled laurel plant *Cryptocarya bourdilloni* [40], which has a unique dihydroflavonoid molecule. Owing to the limited conditions, the Parthasarathy group identified the structure of cryptocaryone (**1**) shown in Figure 2.

In 1985, the correct structure of cryptocaryone (**2**) was determined using a combination of X-ray diffraction, 1D/2D nuclear magnetic resonance (NMR), and previously obtained experimental data [41]. This indicated that the first proposed structure consisted of the functional groups reversed at positions 4 and 9. In addition, it consisted of a cis-conjugated five-membered lactone ring at positions 5 and 6 of the A-ring and was optically active ([*α*]_D_ = + 776.6, *c* = 2, CHCl_3_). Cryptocaryone is the first flavonoid found to contain a chiral A-ring.

Although the relative conformation of cryptocaryone was determined in previous studies, the absolute conformation of positions 5 and 6 remained unresolved. In 2001, the Gueritte group [42] determined the configuration of the two chiral carbons in the (+)-cryptocaryone (**3**) natural product as (5*R*, 6*S*) using bromine substitution of the 8 position cryptocaryone compound followed by X-single crystal diffraction analysis. In 2010, the Kita group [43] obtained (5*R*,6*S*)-cryptocaryone (**3**) and its enantiomeric isomer (5*S*,6*R*)-cryptocaryone (**4**) via the introduction of chiral auxiliary groups. The correctness of the results obtained by Guerette was verified by comparing the spin values obtained from the synthesized product to those of the natural product obtained by isolation.

### 2.2. Infectocaryone, Cryptocaryanones, Bicaryanones, and Chalcocaryanones

**Figure 3:**
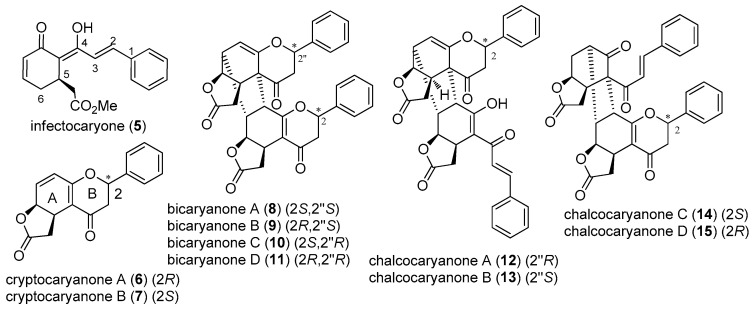
Chemical structure of infectocaryone, cryptocaryanone, bicaryanone, and chalcocaryanone.

After cryptocaryone was reported to contain a chiral A-ring, no other isolation of this flavonoid type was reported for nearly 30 years. However, in 2001, French scientists from the Gueritte group [42] isolated 11 new compounds containing chiral A-ring flavonoids (**5** to **15**) in the bark of a trunk part of a thick-shelled laurel plant *Cryptocarya infectoria* (Figure 3), in addition to the existing cryptocaryone. Infectocaryone (**5**) is a novel chiral A-ring flavonoid-containing molecule, that differs from cryptocaryone in the absence of a five-membered lactone ring and presence of a methyl acetate substituent at position 5. Cryptocaryanone A (**6**) and cryptocaryanone B (**7**), similar to the natural product cryptocaryone, both have chiral lactone rings at positions 5 and 6 of the A ring, excluding the fact that their C3 sites are unchained; however, they form a six-membered oxygen heterocycle (B ring) with the A ring. Bicaryanone A-D (**8–11**) and chalcocaryanone A-D (**12–15**) are the first eight chiral A-ring flavonoid dimers discovered, which are analyzed structurally using polymerization of the chiral A-ring flavonoid monomers through [4 + 2] or [3 + 2] cycloaddition. The structure of this class of natural products was determined using the comparative analysis of infrared, mass spectrometry, and one- and two-dimensional NMR data with that of cryptocaryone.

### 2.3. Desmethylinfectocaryone

**Figure 4:**
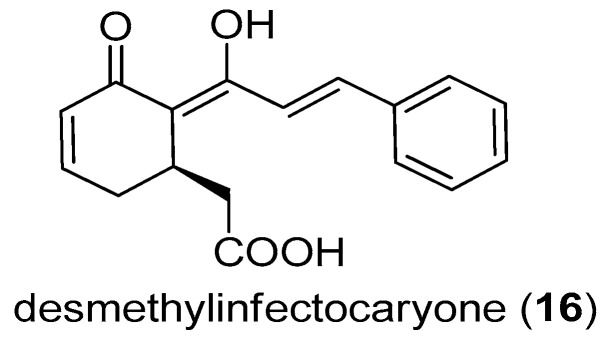
Chemical structure of desmethylinfectocaryone.

A novel chiral A-ring flavonoid-containing compound, desmethylinfectocaryone (**16**, Figure 4), was isolated from the stem of *Cryptocarya Konishi* by F. Kurniadewi [37]. Two known compounds infectocaryone (**5**) and (+)-cryptocaryone (**3**) were isolated simultaneously, and desmethylinfectocaryone (**16**) was a derivative of **5** methyl ester hydrolysis.

### 2.4. Cryptochinones

**Figure 5:**
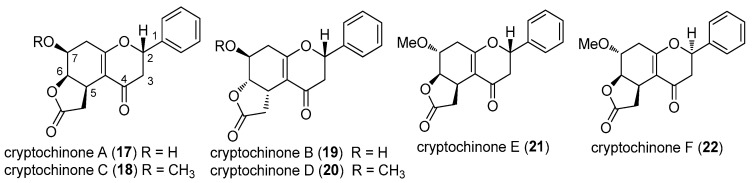
Chemical structure of cryptochinone.

Six novel tetrahydroflavanones cryptochinone A-F (**17–22**, Figure 5) were isolated and extracted from a neutral CHCl_3_ fraction of *Cryptocarya chinensis* leaves [44]. The structures of these new compounds were determined via spectroscopic analyses, including 2D-NMR, MS, CD, and X-ray crystallographic analysis. In contrast to the previously discovered chiral flavonoids with a double bond at C-7, these six natural compounds were flavonoids containing chiral A-rings with C-7 oxo.

### 2.5. Cryptoflavanones

**Figure 6:**
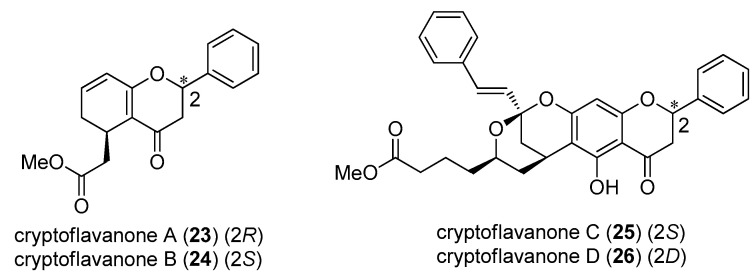
Chemical structure of cryptoflavanones.

Four novel chiral A-ring flavonoids cryptoflavanones A-D (**23–26**) were isolated from the leaves of *Cryptocarya chinensis* [38]. The structures of these four compounds were determined using spectral analyses and are shown in Figure 6.

**Scheme 1:**
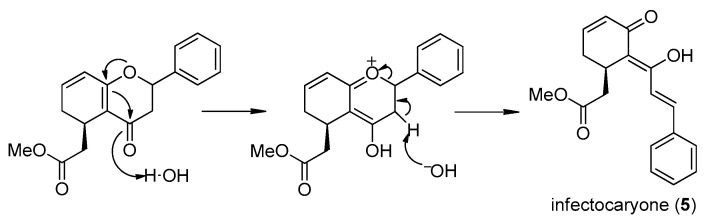
Transformation routes in plants.

The difference between **23** and **24** is observed in the opposite absolute configuration of the two positions, which are a pair of differential isomers and cannot be separated using column chromatography. Furthermore, the peak pattern information on the ^1^H-NMR spectra confirmed that the two compounds were a pair of differential isomers with a ratio of approximately 1.5:1. Though compounds **23** and **24** could be identified separately by the spectral data, they remained practically inseparable from each other. Notably, compounds **23** and **24** can be biotransformed in thick-shelled laurel plants to form the natural product infectocaryone (**5**) shown in Scheme 1.

### 2.6. Cryptogiones

**Figure 7:**
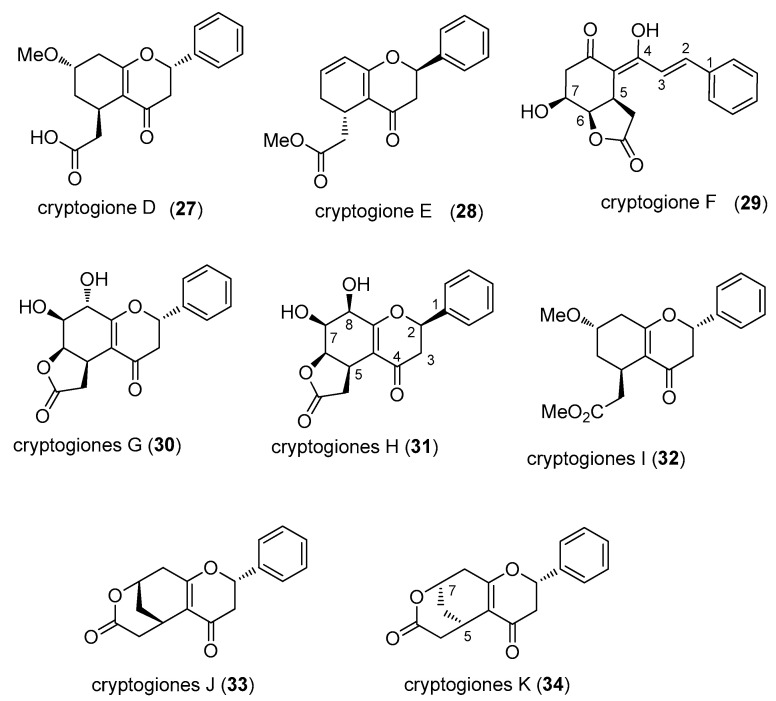
Chemical structure of cryptogiones.

Three novel chiral A-ring flavonoids termed cryptogiones D–F (**27–29**) were isolated from the stem of a thick-shelled laurel plant *Cryptocarya chingii* [45] in 2012. The structures of the three compounds, shown in Figure 7, were elucidated by interpreting the comprehensive spectroscopic data and X-ray analysis.

In 2013, a research group led by Ge [46] obtained five novel chiral A-ring flavonoids cryptogione G-K (**30–34**), from the stem of *Cryptocarya maclurei* (Figure 7). The structures of the isolates were elucidated using 1D and 2D NMR spectroscopic data analysis, and their absolute configurations were determined using CD methods. Compounds **30** and **31** have hydroxyl groups at the seven positions and at the eight positions. Compounds **33** and **34** are able to bridge ring lactones at positions 5 and 7 of the A ring.

### 2.7. Cryptoconones

**Figure 8:**
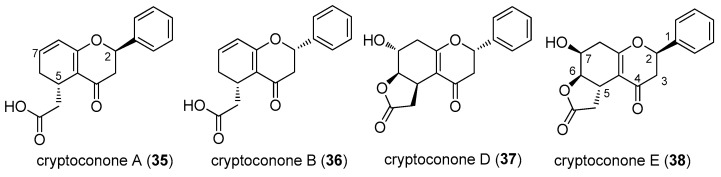
Chemical structure of cryptoconones.

In 2014, the group led by Ge isolated and extracted four novel chiral A-ring-containing flavonoids (**35–38**) from the stem of *Cryptocarya concinna*, a thick-shelled cinnamon plant, and are shown in Figure 8. The structures of these compounds were elucidated on the basis of spectroscopic data interpretation, and the absolute configurations were determined via circular dichroism spectra and X-ray crystal analysis [47]. Moreover, compound **38** is the only chiral flavonoid that a trans-conjugated lactone ring.

Depending on the functional group at position C-7, two types of chiral A-ring flavonoid-containing compounds can be classified as C-7 double bond or C-7 as oxo, and the number of C-7 positions as oxo is higher. Hence, these compounds have only been extracted and isolated from thick-shelled laurel plants, and the isolation of such chiral A-ring flavonoids from other plants have not been reported, which can provide a reference basis for future studies on the classification of natural products.

## 3. Biological Activity of Chiral A-Ring Flavonoid-Containing Compounds

**Figure 9:**
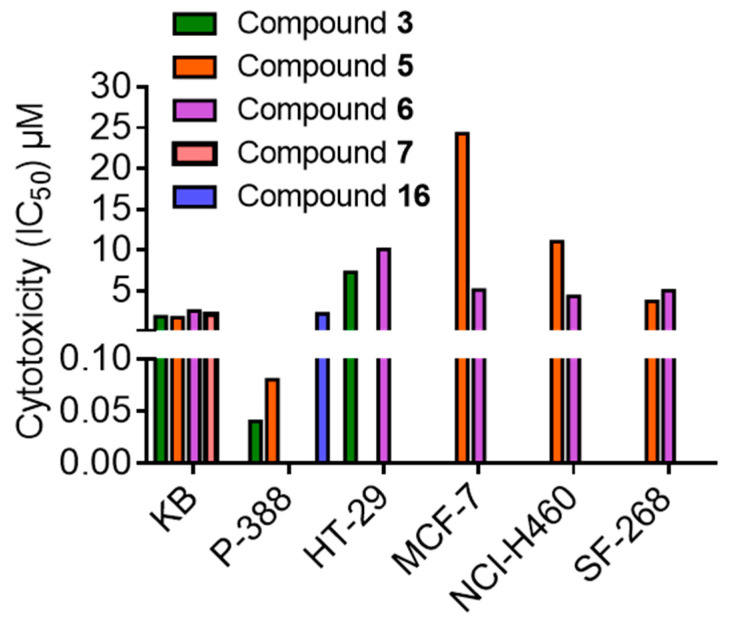
Cytotoxicities of compounds **3**, **5**, **6**, **7** and **16**.

It was shown that these chiral A-ring-containing flavonoids are applicable in various biological activities, and their structure was found to have a strong influence on biological activity (Table 1 and Figure 9).

Gueritte’s group [42] tested the activity of extracted chiral A-ring-containing flavonoid compounds. Activity tests were conducted against human oral epidermal carcinoma cells (KB), human leukemia cells (K562), and adriamycin-resistant cells (K562-DOX) and found that **3**, **5**, **6**, and **7** had good cytotoxicity—in particular, cryptocaryone (**3**), which exhibited good resistance against the different cancer cells. Hence, using these compounds as lead compounds for antitumor drug development is a promising trend [50,51].

In 2010, F. Kurniadewi’s group [37] conducted a study on the cytotoxicity and inhibitory properties of several extracted chiral A-ring flavonoid-containing compounds. It was found that compounds **3**, **5,** and **16** had potent inhibitory effects on murine leukemia cells P-388, with compound **3** displaying good inhibition of tyrosine kinase. Chen et al. [44] conducted cancer cytotoxicity experiments on various extracted chiral A-ring-containing flavonoids extract. Compounds **5** and **6** were found to be active against human breast and lung cancers, and central nervous system cancer cells.

In the same year, Chen et al. [48] performed an in-depth study on the anti-cancer mechanism of compound **3** and found that it induced anti-malignant cell proliferation and affected apoptosis in humans and exhibited good inhibitory activity against male prostate cancer cells. In addition, it caused cysteine protease (caspase-8 and 3) activity; however, it did not alter the total protein content of the death receptors and ligands.

In 2011, Chen et al. [38] performed cytotoxicity assays with **3** against the mycobacterium tuberculosis (*H_37_Rv*) strain and observed some activity when the minimum inhibitory concentration (MIC) was 25.0 μg/mL.

Ge et al. [45] conducted activity experiments in 2012 on extracted chiral A-ring flavonoid-containing compounds and observed that all the extracted compounds had anti-inflammatory properties. Compounds **3** and **7** were pretreated to completely block the tumor necrosis factor (TNF)-induced degradation of the nuclear factor inhibitor protein (IB). This indicates that both compounds exhibited inhibition of the tumor necrosis factor (TNF) activity to induce inflammatory responses, with an inhibitory activity IC_50_ of 2.0 μM. In addition, they observed that **3** and **7** exhibited good inhibitory activity against bacterial-induced inflammation.

Ge et al. [46] reported that compound **3** exhibited moderate cytotoxicity against human tumor cell lines (HCT116), human colon cancer cell lines (SW480), human colon cancer cells (HT-29), and human breast cancer cells (MDA-MB-231) with an IC_50_ range of 6.25–9.35 μM. Moreover, compound **6** displayed antibacterial activity against fumonisins and Staphylococcus griseus, among others.

In 2014, Kinghorn et al. [39] performed cytotoxicity assays against human colon cancer cells (HT-29) and observed that compound **3** exhibited good inhibitory activity (IC_50_ = 0.32 μM), and compound **6** had moderate inhibitory activity (IC_50_ = 10.9 μM). The structures of compounds **3** and **6** were compared, and it was concluded that the extensive conjugation system of **3** was favorable for its inhibitory activity. Furthermore, they found that **3** has an inhibitory effect on glucose transportation.

Chen et al. [52] investigated the activity of extracted compounds and found that compounds **17** to **20** are structurally similar to the non-steroidal farnesylate X receptor FXR ligands and therefore could act as a target. FXR is a member of the nuclear receptor family, which is at common target for the treatment of chronic liver diseases and hyperglycemia. In addition, compounds **17** to **20**, in which C-7 is oxo and can invert the activation of FXR receptors and act as promoters of the transcriptional activator protein (GAL4), cholesterol 7-hydroxylase (CYP7A1), and phospholipid transfer protein (PLTP).

Chang et al. [53] found that compounds **3** and **5** can reduce the production of oral cancer cells such as Ca9-22 and CAL27. The compounds induce oral cancer cell generation, apoptosis, oxidative stress, and DNA damage; hence, they are considered as a potential natural product for the reactive oxygen species-mediated treatment of oral cancer. They also reported that the combined ultraviolet C (UVC) and cryptocaryone (CPC) exhibited higher anti-proliferation than individual and control treatments in a low cytotoxic environment on normal oral cells. UVC/CPC treatment in combination causes higher oxidative stress, G2/M cell cycle arrest, DNA damage, and apoptosis in oral cancer cells [54].

Recently, Chang [49] performed cytotoxicity assays against three histotypes of ovarian cancer (OVCA) cells (TOV-21G, SKOV3 and TOV-112D) and observed that compound **3** exhibited good inhibitory activity (IC_50_ = 1.5, 3 and 9.5 μM). They found that compound **3** was a potential anti-OVCA natural product showing oxidative stress-dependent antiproliferation, apoptosis, and DNA damaging functions.

## 4. Progress in the Synthesis of Chiral A-Ring-Containing Flavonoids

Due to the unique structure of chiral A-ring-containing flavonoids and several antitumor and antibacterial activities, their interest for use in synthesis has increased. Although these compounds were discovered earlier, most were not taken seriously until after 2001. The activity studies were performed relatively late; hence, minimal synthetic work has been conducted. Currently, only four reports have described the synthesis of chiral A-ring-containing flavonoids. Their artificial work will be described separately in the following subsections.

### 4.1. Synthesis of the Natural Product Cryptocaryone

In 2010, the group led by Kita [43] synthesized the natural product (+)-cryptocaryone and its enantiomer (−)-cryptocaryone, in a nine-step reaction with 5.9% overall yield employing 1,4-cyclohexadiene as the initial raw material. They used a strategy induced by the introduction of a chiral auxiliary group.

**Scheme 2:**
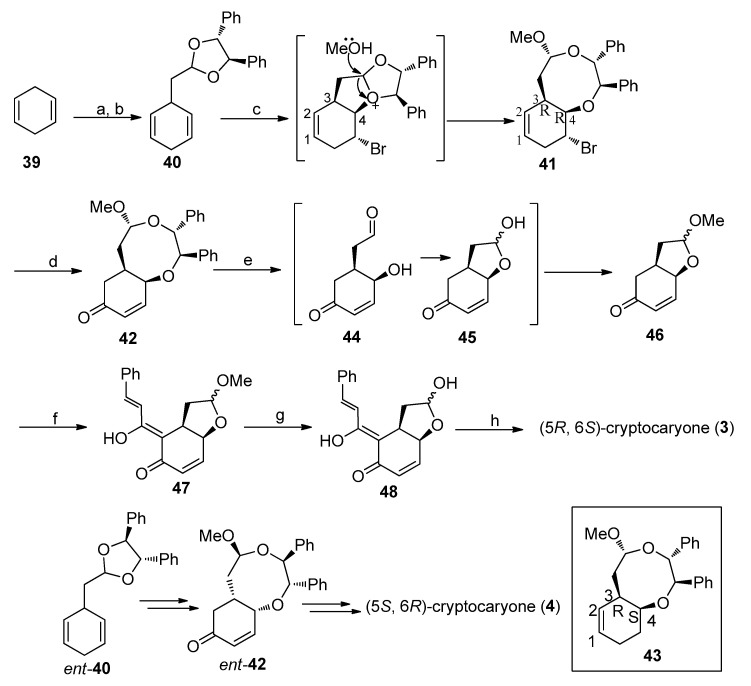
Synthesis of the natural product (+)-cryptocaryone and its enantiomeric isomers. Conditions: (a) *s*-BuLi, TMEDA, THF, −78 °C, then BrCH_2_CH(OEt)_2_; (b) (*R*,*R*)-hydrobenzoin, *p*-TsOH, toluene, 50 °C, 75% for two steps; (c) NBS, CH_3_CN, 63%; (d) thexylborane, then PDC, 53%; (e) CAN, CH_3_CN-H_2_O (2/1), 60 °C, 67%; (f) LiHMDS, PhCH=CHCOCl, THF, −78 °C; (g) 4 N HCl aq., 64%; (h) NIS, TBAI, DCM, 74%.

As shown in Scheme 2, the acetals were obtained first using 1,4-cyclohexadiene as the starting material and subsequently introduced chiral auxiliary groups by interacting with (*R*, *R*)-diphenylethylene glycol to form acetals. Subsequently, the chiral cyclic acetals underwent a bromonium ion-initiated ring expansion reaction in the presence of methanol in the NBS to obtain octaheterocyclic compound **41** containing multiple chiral centers such as bromine. Next, borohydride oxidation reaction and elimination of Br atoms was performed to obtain cyclohexenone acetals compound **42** containing chiral auxiliary groups. To determine the absolute configuration of the chiral center, combination **43** was received by reducing the Br atom of compound **41** and then the fundamental structure of compound **43** at positions 3 and 4 were selected using X-single crystal diffraction and NOESY data as (3*R*,4*S*), which leads to absolute configuration in **41** as (3*R*,4*R*) [55].

The aldehyde **44** is then obtained by the acetal hydrolysis [56,57,58] of **42** and removal of the diphenyl glycol group [59,60], that will spontaneously become hemiacetal **45**. Since hemiacetal **45** is unstable and can decompose and deteriorate when concentrated or separated by column chromatography using silica gel, a one-pot reaction was used to obtain acetal **43** by directly treating the mixture containing **44** with methanol. After obtaining compound **46**, the ketone α-H was removed using a strong base LiHMDS, and subsequently condensation with cinnamoyl chloride occurred to obtain enol compound **47.** Next, the hydrolysis by hydrochloric acid was performed to obtain hemiacetal **48.** Finally, the compound (5*R*,6*S*)-cryptocaryone (**3**) was oxidized in 74% yield using NIS and TBAI as oxidizing reagents [61].

Following the same synthetic process, 1,4-cyclohexadiene was used as the starting material, and (*S*,*S*)-diphenyl glycol was used as a chiral auxiliary group to synthesize the cyclohexadiene acetal compound ent-**40**, which resulted in enantiomeric ent-**42**. Compound (5*S*,6*R*)-cryptocaryone (**4**) was finally synthesized using the same process.

After obtaining compounds **3** and **4**, the spins were determined separately, and the absolute configuration of the natural product (+)-cryptocaryone was verified as (5*R*,6*S*) by comparing with the spin values of the natural product.

### 4.2. Synthesis of the Natural Products (+)-Cryptocaryone and (+)-Infectocaryone

Helmchen’s group [62] completed a total synthesis of the chiral A-ring flavonoid-containing compounds (+)-cryptocaryone (**3**) and (+)-infectocaryone (**5**) in 32% and 26% overall yields, respectively. The key steps of the synthesis included starting with the carbonate of protected allyl using metal Ir-catalyzed asymmetric alkylation at the allyl position and the RCM reaction.

**Scheme 3:**
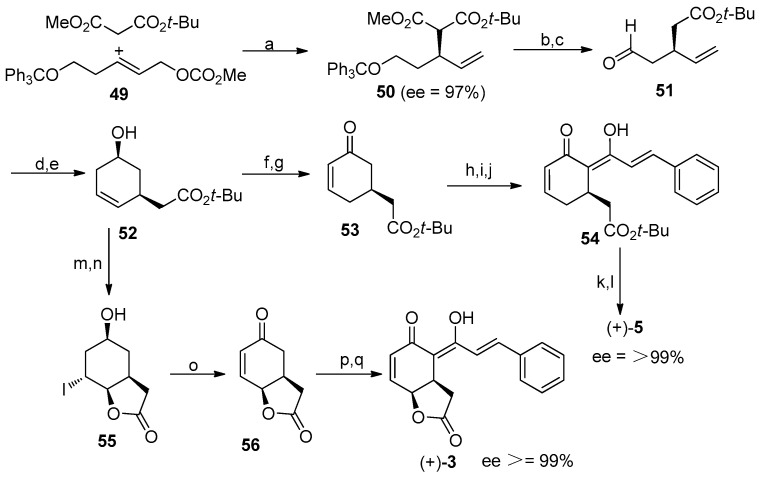
Synthesis of the natural products (+)-cryptocaryone and (+)-infectocaryone. Conditions: (a) [Ir]/L catalyst, 85%; (b) NaOH, DMF, 155 °C, 82%; (c) AcOH, then DMP, 82%; (d) (+)-(Ipc)_2_B(allyl), Et_2_O, −100 °C; (e) Grubbs’I, DCM, 40 °C, 81%; (f) Dess-Martin oxidation; (g) DBU, 97%; (h) *n*-Bu_2_BOTf, DIPEA, −78 °C; (i) cinnamaldehyde; (j) Dess-Martin oxidation, 64%; (k) TFA/DCM; (l) CH_2_N_2_, 91%; (m) NaOH, 90 °C; (n) NaHCO_3_, KI, I_2_, THF, H_2_O; (o) Swern oxidation, 75%; (p) LiHMDS, THF, −100 °C; (q) cinnamoyl cyanide, 74%.

As shown in (Scheme 3), compound **50** was synthesized in 85% yield and up to 97% enantiomeric excess (*ee*) using the allyl-site asymmetric alkylation reaction system [63,64,65] developed by the group (catalyst [Ir(dbcot)Cl]_2_, L* as chiral ligand, and TBD as the base) with protected allyl carbonate **49** and malonate as raw materials. Subsequently, the selective hydrolysis of methyl ester, heating decarboxylation, acetic acid removal of the triphenylmethyl protection, and Dess–Martin oxidation resulted in aldehyde 2-**48**. Finally, the aldehyde group in **51** was attacked using a chiral borane reagent to form a chiral high allyl alcohol compound and RCM ring closure reaction in the presence of a Grubbs generation catalyst to give advanced intermediate **52**.

After intermediate **52** was obtained, the synthesis of the natural product (+)-infectocaryone (**5**) was attempted. The Dess-Martin reagent was employed to oxidize the hydroxyl group of **52**, followed by double bond rearrangement in the presence of DBU to obtain the conjugated α,β-unsaturated ketone **53**. Compound **53** underwent aldol reaction with trans-cinnamaldehyde in DIPEA and *n*-Bu_2_BOTf [66,67], and Dess-Martin oxidation to obtain **54** in 64% yield. Finally, the natural product (+)-**5** was obtained through the hydrolysis of the ester in the presence of TFA and methylation in diazomethane.

After completion of the synthesis of the natural product (+)-**5**, the synthesis of (+)-**3** was attempted. First, the ester group in compound **52** was subjected to alkaline hydrolysis and lactonization in the presence of iodine monomers to obtain **55**. Swern oxidation, under which **55** was converted to ketones using simultaneous elimination of HI showed the unsaturated ketone **56** and the structure was determined using X-single crystal diffraction. Next, the natural product (+)-**5** was synthesized and unable to undergo condensation with trans-cinnamaldehyde. Hence, a selective C-acylation reaction was performed directly on **56** to obtain the natural product (+)-**3**. After screening the acylation reagents and bases several times, the total synthesis of the natural product (+)-**3** was finally completed in good yield using lithium hexamethyl disilyl amine as the base at −100 °C and cinnamoyl cyanide as the acylation reagent.

Although expensive catalysts and harsh reaction conditions were employed in some steps, the process remains a good future reference for the synthesis of natural products owing to their high stereoselectivity and overall yields.

### 4.3. Asymmetric Synthesis of the Natural Product Cryptocaryanone A

**Scheme 4:**
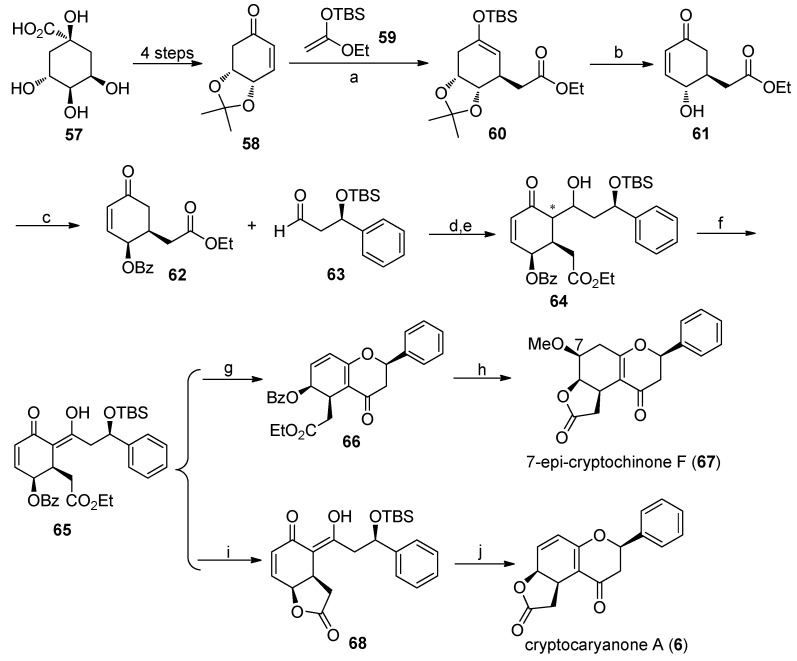
Synthesis of natural product cryptocaryanone A. Condition: (a) cat. HgI_2_, Et_2_O, −78 °C to rt, 90%; (b) NaOH (0.5 M), TBAF, THF, 0 °C, 85%; (c) Ph_3_P, PhCO_2_H, DIAD, THF, 0 °C to rt; 82%; (d) TBSOTf, Et_3_N, DCM, 0 °C to rt; (e) BF_3_·OEt_2_, DCM, −78 °C, 72% for 2 steps; (f) TFAA, DMSO, Et_3_N, DCM, −78 °C, 87%; (g) TfOH, DCM, −30 °C, 91%; (h) K_2_CO_3_, MeOH, rt, 30%; (i) K_2_CO_3_, MeOH, rt, 61%; (j) TsOH, DCM, rt, 49%.

In 2012, a research group led by She [68] first synthesized natural product cryptocaryanone A (**6**) starting from the chiral raw material d-(−)-quinic acid, using the Mukaiyama aldol reaction and the one-pot cyclization reaction as the key steps. In addition, the compound 7-epi-cryptochinone F (Scheme 4) was obtained.

First, using the chiral raw material d-(−)-quinic acid (**57**), the chiral unsaturated ketone **58** was obtained in a four-step reaction reported in the literature [69,70], followed by Michael addition with compound **59** using HgI_2_ as catalyst [71] to obtain ester **60** in 90% yield. This was followed by obtaining allyl alcohol compound **61** using TBS protection and elimination [72], and cyclohexenone **62** with hydroxyl configuration flip using the Mitsunobu reaction.

After obtaining compound **62**, selective p-keto carbonyl enolization yielded enol silyl ether, followed by Mukaiyama aldol reaction with aldehyde **63** catalyzed using BF_3_-Et_2_O to obtain compound **64**, followed by Swern oxidation [73] to obtain enol compound **65**. Two processes were attempted to determine whether to first turn off the B ring or lactone ring. First, compound **65** was deprotected from TBS in the presence of TfOH and underwent intramolecular cyclization in a one-pot method to obtain product **66** with the B ring off. Finally, the lactonization reaction of **66** did not yield cryptocaryanone A. However, a novel compound was obtained with a 30% yield, and this novel compound was identified as 7-epi-cryptochinone F (**67**) using combined analysis of one- and two-dimensional NMR data.

Because the natural product was not obtained by turning off the B ring first, it was then decided that the lactone ring should be turned off first followed by the B ring. Threfore, adding potassium carbonate to the methanol solution of **65** resulted in lactone compound **68**. Then, natural product **6** was obtained through one-pot reaction in the presence of TsOH at 49% yield.

### 4.4. Asymmetric Synthesis of Infectocaryone

Chen’s group [74] synthesized the natural product (+)-infectocaryone (**5**) and its enantiomeric isomers using the Diels-Alder reaction as the critical step and 2-deoxy-D-ribose as the initial raw material.

**Scheme 5:**
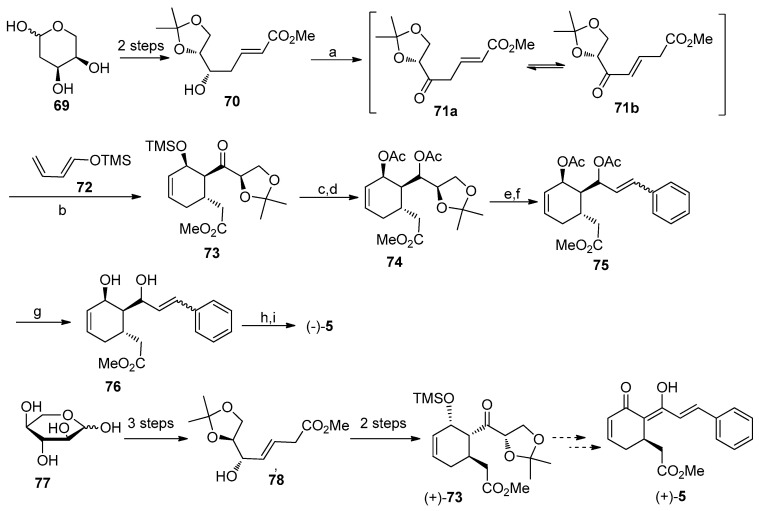
Synthesis of (−)-infectocaryone and its enantiomeric isomers. Condition: (a) IBX, AcOEt, 80 °C; (b) toluene, 120 °C, 50% for 2 steps; (c) NaBH_4_, MeOH, rt, 88%; (d) Ac_2_O, DMAP, DCM, rt, 96%; (e) H_5_IO_6_, AcOEt, rt, 99%; (f) [Ph_3_PCH_2_Ph]·Br, *n*-BuLi, THF, 75%; (g) K_2_CO_3_, MeOH, H_2_O, rt, 71%; (h) DMP, DCM, rt; (i) Pd(PPh_3_)_4_, THF, rt, 92% for two steps.

As shown in Scheme 5, starting from the chiral raw material 2-deoxy-*D*-ribose, an unsaturated ester **70** was obtained through a two-step reaction, followed by the oxidation of the hydroxyl group using IBX to obtain the dienophile; however, because the double bonds of the oxidation products were in dynamic equilibrium, two dienophile mixtures **71a** and **71b** were obtained. After obtaining the dienophiles, the Diels-Alder reaction with the diene **72** was attempted and a significant cycloaddition product **73** was obtained in a two-step 50% yield. This indicated a higher activity of the dienophile whose double bond is conjugated to the ketone carbonyl group, mainly via the reaction of **71b** with the diene. To determine the absolute configuration of the newly generated chiral center, the double bond of the Diels-Alder reaction product **73** with Pd/C was reduced and subsequently removed the TMS protection to obtain secondary alcohol that reacted with (*S*) and (*R*)-(−)-α-methoxyphenylacetic acid, respectively. The absolute configuration of the secondary alcohol was determined according to the Mosher model derivatization method developed by Trost [75,76,77]. Finally, the complete structure of compound **73** was determined by combining the NMR data.

Combination **74** was obtained by reduction using sodium borohydride and protection of hydroxyl groups by acetyl groups, followed by oxidation with periodate to remove the ketone-protected o-diol and obtain an aldehyde. The Wittig reaction was employed next to obtain compound **75** containing a double bond, and the removal of acetyl groups to obtain alcohol **76**, and finally oxidation of alcoholic hydroxyl groups and isomerization to obtain the final product (−)-**5**.

After enantiomer of the natural product was obtained, the chiral raw material with L-xylose was replaced to obtain compound 78 in a three-step reaction, followed by the oxidation of the hydroxyl group to obtain the enantiomer (*S*)-71a/b, and subsequently the Diels-Alder reaction with diene 72 to obtain the enantiomer (+)-73. The synthesis of the natural product (+)-5 can be completed using the same synthetic process.

### 4.5. Asymmetric Synthesis of Cryptogione F, Cryptocaryanone B, Cryptochinones A and C, Cryptocaryone, and Cryptocaryanone A

In 2018, Chen’s group [78] synthesized (+)-cryptogione F, (+)-cryptocaryanone B, (+)-cryptochinones A and C, (+)-cryptocaryone, and (+)-cryptocaryanone A using the coupling via a boron-mediated aldol condensation and the cyclization via a highly stereoselective intramolecular Michael addition of 1,3-diketone processed under mild conditions.

**Scheme 6:**
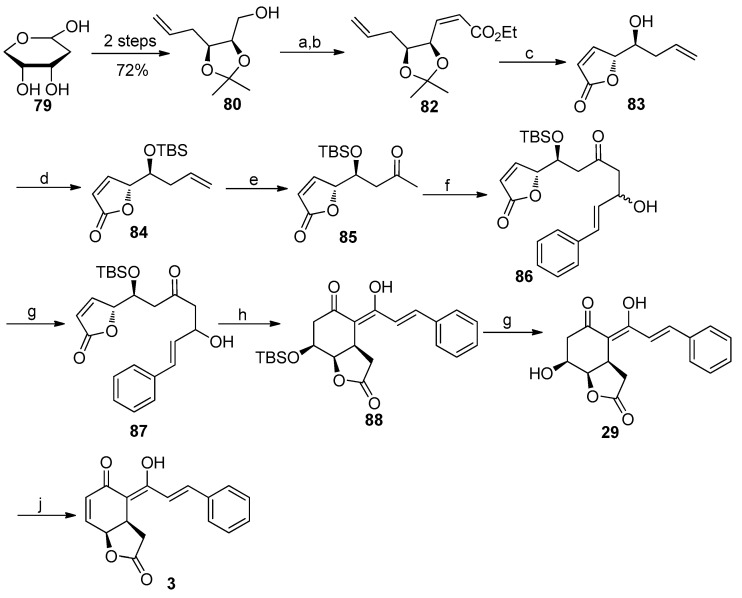
Synthesis of (+)-cryptogione F and (+)-cryptocaryone. Condition: (a) Swern oxidation; (b) 2) **81**, MeOH, 0 °C, 80% for two steps; (c) *p*-TsOH, MeOH, rt, 96%; (d) TBSCl, imidazole, DMF, rt, 97%; (e) O_2_, CuCl, PdCl_2_, DMF/H_2_O, 84%; (f) Cy_2_BCl, Et_3_N, Et_2_O, −78 °C, cinnamaldehyde, 85%; (g) DMP, DCM, rt, 70%; (h) DBU, DCM, 83%; (i) HF·Py, MeCN, 84%; (j) Burgess reagent, 71%.

As shown in Scheme 6, the synthesis started with the preparation of chiral ketone from 2-deoxy-*D*-ribose **79**. The terminal alkene **80** was obtained using a two-step reaction as reported in the literature [79,80]. Swern oxidation of primary alcohol **80** resulted in the corresponding aldehyde, followed by Wittig reaction with (carbethoxymethylene)-triphenylphosphorane **81** to obtain *Z*-conjugated ester **82** in good yield. Following the literature [81], in the presence of *p*-toluenesulfonic acid, the one-pot method removed the ketal protection and ester exchange occurred to form unsaturated lactone **83**, and the free hydroxyl group was protected by TBS to give terminal olefin **84**. Wacker oxidation of the terminal double bond of **84** yielded methyl ketone **85**, which was treated with chlorodicyclohexylborane and reacts with cinnamaldehyde in an aldol reaction to give a mixture of diastereoisomeric adducts **86**. The mixture was oxidized using Dess-Martin to give enol ketone **87**, followed by an intramolecular Michael addition reaction under milder reaction conditions to give **88**. The silyl group of **88** was removed smoothly using HF·Py to give (+)-cryptogione F (**29**). Further β-elimination of the hydroxyl group in **29** with Burgess reagent [82,83] furnished (+)-cryptocaryone (**3**).

**Scheme 7:**
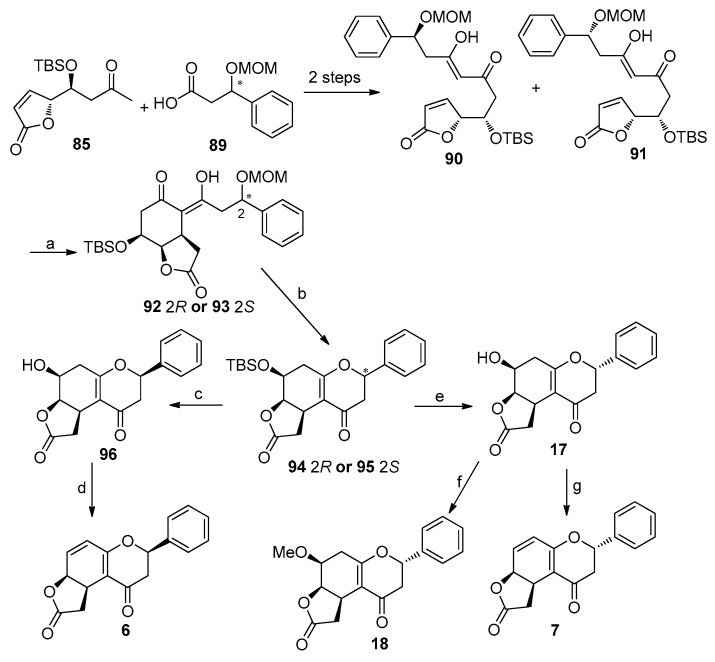
Synthesis of (+)-cryptocaryanone A, (+)-cryptochinone A, (+)-cryptochinone C and (+)-cryptocaryanone B. Condition: (a) DBU, DCM, −20 °C; (b) TFA, DCM, rt, 88% for **94**, 89% for **95**; (c) HF·Py, MeCN, 87% from **94**; (d) MsCl, Et_3_N, DCM, rt, 93%; (e) HF·Py, MeCN, 86% from **95**; (f) MeI, Ag_2_O, CaSO_4_, rt, 92%; (g) MsCl, Et3N, DCM, rt, 90%.

Following the same procedure used for the synthesis of **86**, chiral aldehydes (*R*)- and (*S*)-**89** were converted to enolones **90** and **91**, respectively. Construction of the B-ring was achieved by TFA mediated cleavage of the MOM protecting group in **92**, concomitant formation of hemiacetal, and dehydration to produce hydropyranone (**94**). After deprotection of the TBS group, the resulting alcohol (**95**) was subjected to one-pot sulfonylation and elimination to furnish (+)-cryptocaryanone A (**6**). The other adduct (**95**) was transformed to (+)-cryptochinone A (**17**), which underwent *O*-methylation to give (+)-cryptochinone C (**18**). Moreover, (+)-cryptocaryanone B (**7**) was obtained from the treatment of **17** with MsCl and Et_3_N (see Scheme 7).

## 5. Conclusions

In this review, the recently discovered chiral A-ring-containing flavonoids were listed and their biological activities together with their synthetical approaches were summarized. With the recent increase in new members of this group of natural products and the gradual development of research on their mechanism of activity, they have been identified as essential in the medical field. Therefore, the development of efficient, simple, and universal synthetic routes to obtain a series of these flavonoids and derivatives is of good theoretical value in synthetic chemistry and has a positive impact on the prospect of new drug development for these compounds, which is of great importance to several fields.

## Figures and Tables

**Table 1:** Bioactivities of all novel chiral A-ring flavonoid-containing compounds.

Compound	Cytotoxicity (IC_50_) µM [Cell Line]	Ref.
**3**	1.8 [KB], 2.0 [K562], 2.0 [K565-DOX], 0.04 [P-388], 1.6 [PC-3], 2.3 [DU-145], 3.4 [LNCaP], 9.35 [HCT116], 7.23 [HT-29], 6.52 [SW480], 9.03 [MDA-MB-231], 0.32 [HT-29], 1.5 [TOV-21G], 3 [SKOV3], 9.5 [TOV-112D]	[37,39,42,47,48,49]
**5**	1.7 [KB], 0.08 [P-388], 24.3 [MCF-7], 11.0 [NCI-H460], 3.7 [SF-268]	[37,42,44]
**6**	2.5 [KB], 10.9 [HT-29], 5.1 [MCF-7], 4.3 [NCI-H460], 5.0 [SF-268]	[39,42]
**7**	2.1 [KB]	[42]
**16**	2.17 [P-388]	[37]
Antimicrobial activity (MIC; µg/mL)		
**3**	25 [*H_37_Rv*]	[44]
**6**	10 [*Phytophthora capsici*], 5 [*Fusarium moniliforme*]	[47]
Anti-inflammatory activity (IC_50_) µM		
**2**	2.0 [TNFa]	[46]
**7**	2.0 [TNFa]	[46]

## Data Availability

Not applicable.

## Abbreviations

CAN	Ceric ammonium nitrate
DBU	1,8-Diazabicyclo [5.4.0]undec-7-ene
DCM	Dichloromethane
DMAP	4-Dimethylamino-pyridine
DMF	Dimethyl formamide
DMP	Dess-Martin periodinane
IBX	2-Iodoxybenzoic acid
IC_50_	Half maximal inhibitory concentration
LiHMDS	Lithium bis(trimethylsilyl)amide
NBS	*N*-Bromosuccinimide
NIS	*N*-iodosuccinimide
PDC	Pyridinium dichromate
RCM	Ring-closing metathesis
TBAI	Tetrabutylammonium Iodide
TFAA	Trifluoroacetic anhydride
THF	Tetrahydrofuran
TMEDA	Tetramethylethylenediamine
